# Characterizing and quantifying the temporal relationship between structural and functional change in glaucoma

**DOI:** 10.1371/journal.pone.0249212

**Published:** 2021-04-01

**Authors:** Fang-I Chu, Lyne Racette

**Affiliations:** 1 Eugene and Marilyn Glick Eye Institute, Indiana University, Indianapolis, IN, United States of America; 2 Department of Radiation Oncology, University of California, Los Angeles, CA, United States of America; 3 Department of Ophthalmology and Visual Sciences, University of Alabama at Birmingham, Birmingham, AL, United States of America; Bascom Palmer Eye Institute, UNITED STATES

## Abstract

**Purpose:**

To characterize and quantify the temporal relationship between structural and functional change in glaucoma.

**Methods:**

120 eyes of 120 patients with ocular hypertension or primary open-angle glaucoma were selected from the Diagnostic Innovations in Glaucoma Study or the African Descent and Glaucoma Evaluation Study. Patients had 11 visits, separated by at least 3 months over 5 to 10 years. Each visit had rim area (RA) and mean sensitivity (MS) measurements taken within a 30-day period. The structure-function (SF) relationship was summarized using conventional and modified cross-correlation functions (CCFs), which identified the strongest absolute and positive correlation, respectively. Patients were categorized in one of the following three groups: RA and MS evolved simultaneously (lag = 0), RA preceded MS (lag<0), and MS preceded RA (lag>0). Lagging regression analysis was used to examine the variations of the SF relationship within groups.

**Results:**

The number of participants, mean visit lag, and mean correlation (standard deviation) were, for the conventional and modified CCFs, respectively: lag = 0 [16, 0, 0.53 (0.10) and 16, 0, 0.46 (0.11)]; lag<0 [50, −2.94, 0.51 (0.11) and 55, −3.45, 0.44 (0.12)], and lag>0 [54, 3.35, 0.53 (0.13) and 49, 3.78, 0.45 (0.12)]. A significant difference of the visit lag relation within groups was identified using lagging regression analysis (p<0.0001).

**Conclusions:**

The strongest relationship between structure and function was obtained at different visit lags in different patients. This finding also suggests that the SF relationship should be addressed at the subject level when using both measurements jointly to model glaucoma progression.

## Introduction

Primary open-angle glaucoma (POAG) is a chronic optic neuropathy in which the progressive degeneration of retinal ganglion cells and their axons is associated with visual field loss. While the disease involves changes in both structure and function, several clinical studies have reported relatively weak cross-sectional correlations between structural and functional measurements [1–4]. Efforts made to improve the strength of the structure-function relationship have resulted in only modest improvements. Nassiri et al. [5] assessed the longitudinal relationship between structure and function by correlating series of structural and functional measurements. While correlations reached statistical significance in some sectors, the strength of the correlations were overall weak, with the Spearman correlation coefficients ranging from -0.07 to 0.39.

The relatively weak associations reported between structure and function could be explained, at least in part, by the temporal relationship between structural and functional change in glaucoma. While it is often asserted in the literature that the detection of structural degeneration precedes the detection of visual field loss in the development of glaucoma, there is evidence to suggest that this order is reversed in some patients [6–10]. Studies have shown that progression can be present in a series of visual field tests and be absent on structural data taken during the same follow-up period. Similarly, findings from the Ocular Hypertension Treatment Study (OHTS) [11] and European Glaucoma Prevention Study (EGPS) [12] indicate that not all patients reach the structural endpoint first. This suggests that there may not only be a time lag between the detection of structural and functional defects, but that this lag can favor either structural or functional measurements in different patients. The goal of this study was to characterize and quantify the temporal relationship between structural and functional measurements.

## Methods

The study was approved by the Institutional Review Board at Indiana University and at the University of Alabama at Birmingham. The DIGS and ADAGES studies were approved by the Institutional Review Boards at each of the three sites at which it was conducted (University of California San Diego, New York Eye and Ear Infirmary, and the University of Alabama at Birmingham). Written consent was obtained from each participant. These multicenter studies adhered to the tenets of the declaration of Helsinki for research involving human subjects, and were performed in conformity with the Health Insurance Portability and Accountability Act (HIPAA).

This study included 120 eyes of 120 subjects who were selected from the Diagnostic Innovations in Glaucoma Study (DIGS) and the African Descent and Glaucoma Evaluation Study (ADAGES). The selection of subjects and analysis of this study were performed retrospectively. The descriptive statistics of the demographic features and clinical measures at baseline are summarized in Table 1. The DIGS/ADAGES studies are ongoing longitudinal studies designed to assess structure and function in glaucoma and have been described in detail elsewhere [13].

**Table 1:** Descriptive statistics of demographic features and clinical measures at baseline.

Demographic features*		
Number of subjects = 120	Mean(sd)/ count(%)	Median (IQR, range)**
Age (year)	58.7 (9.7)	58.0 (14, 34–82)
Gender		
Male	57 (47.5%)	
Female	63 (52.5%)	
Race		
Asian	1 (0.8%)	
Black or African American	70 (58.3%)	
Chinese	1 (0.8%)	
White	48 (40%)	
Clinical measures at baseline		
MS (dB)	27.7 (4.6)	29.1 (3.2, 5.1–32.6)
MD (dB)	-2.7 (4.6)	-1.1 (3.0, -25.6–1.9)
RA global (mm^2^)	1.3 (0.4)	1.2 (0.4, 0.3–2.2)
RA ST(mm^2^)	0.2 (0.1)	0.2 (0.1, 0.0–0.3)
RA IT(mm^2^)	0.2 (0.1)	0.2 (0.1, 0.0–0.4)
MRNFL global (μm)	0.2 (0.1)	0.2 (0.1, 0.1–0.5)
MRNFL ST(μm)	0.3 (0.1)	0.2 (0.1, 0.1–0.7)
MRNFL IT(μm)	0.2 (0.1)	0.2 (0.1, -0.2–0.5)
Disc Area global (mm^2^)	2.2 (0.6)	2.1 (0.7, 1.0–3.9)

* Continuous variables are presented as mean (standard deviation) and categorical variables are presented as count (percentage of entire cohort).

**(interquartile range, minimum–maximum).

In the DIGS/ADAGES studies, all participants underwent a comprehensive ophthalmic examination, including review of medical history, best-corrected visual acuity, slit-lamp biomicroscopy, intraocular pressure (IOP) measurement, gonioscopy, and dilated funduscopic examination. The studies required at least one good-quality stereoscopic photograph and one reliable static automated perimetry (SAP) at baseline. All participants had open angles, best-corrected acuity of 20/40 or better, spherical refraction within 5.0 diopters, and cylinder correction within 3.0 diopters. Participants were excluded if they had a history of intraocular surgery (except for uncomplicated cataract surgery); secondary causes of glaucoma (e.g., iridocyclitis, trauma); other systemic or ocular diseases known to affect the visual field (e.g., pituitary lesions, demyelinating diseases, human immunodeficiency virus positive or acquired immune deficiency syndrome, or diabetes); medications known to affect visual field sensitivity; an inability to perform visual field examinations reliably; or life-threatening diseases.

### Inclusion criteria for the present study

Our study included participants with ocular hypertension (OHT, 33 eyes, 27.5%) or primary open-angle glaucoma (POAG, 87 eyes, 72.5%). At the first visit, among the POAG eyes, 30 eyes (25.0%) had glaucomatous optic neuropathy only; 23 eyes (19.2%) had abnormal visual field test result only, while 34 eyes (28.3%) had both abnormal visual field test result and glaucomatous optic neuropathy. The classification criteria for each category have been reported by Sample et al. [13]. All patients who had 11 visits over a period of 5 to 10 years in DIGS/ADAGES dataset were included. A visit was composed of paired structural and functional tests taken within a 30-day period. Visits were separated by at least 3 months. When both eyes from the same patient were eligible, we randomly selected one eye to eliminate the within-subject correlation from paired eyes.

### Structural and functional tests

#### Structural measures

Rim area (RA) was obtained by confocal scanning laser ophthalmoscopy with the Heidelberg Retina Tomograph II (HRTII, software version 3.1; Heidelberg Engineering, Heidelberg, Germany). The HRT software acquires three individual images for each eye during the initial scanning, from which it automatically computes a mean topography image. An experienced technician outlined the optic disc margin on the mean topography image while viewing simultaneous stereophotographs of the optic disc. Only the images with mean pixel height standard deviation less than 50 μm were used, based on the recommendation of the manufacturer [14].

#### Functional measures

We included the results from SAP tests taken with the 24–2 pattern and Swedish interactive thresholding algorithm [15] on the Humphrey Field Analyzer (Carl Zeiss Meditec, Dublin, CA, USA). All visual fields were evaluated by the Visual Field Assessment Center at the Department of Ophthalmology, University of California-San Diego [16]. Visual fields with more than 33% fixation losses, false-negative responses, or false-positive responses, were considered unreliable and were excluded. Visual fields with artifacts (e.g., lid and lens rim artifacts, fatigue effects) were also excluded. The two locations above and below the blind spot were excluded. The sensitivities in decibels (dB) at each of the remaining 52 test locations were first converted to the linear scale in 1/Lambert (1/L) [17, 18] and then averaged to obtain the mean sensitivity (MS) value.

#### Conversion of units for structural and functional measurements

Before assessing the longitudinal relationship between the series of measurements for RA and MS, we transformed the units of both into a comparable scale. RA and MS were converted and expressed in percent of mean normal [19, 20] based on an independent dataset of 91 eyes from 91 healthy subjects [21]. Mean normal values were 1.44 mm^2^ for RA and 1112.63 1/L for MS. The percent of mean normal was computed by scaling the values obtained for each eye using this sample of normal eyes [21] as a reference. It should be noted that the values after the conversion could be larger than 100% for some eyes.

### Statistical analysis

**Fig 1:**
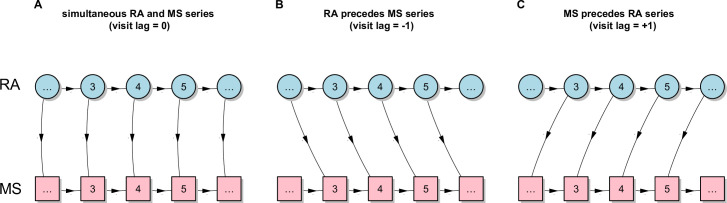
A graphical illustration of the three possible scenarios derived by CCF. Panel A shows the two series of data that evolve simultaneously, panel B shows a series of RA data that precedes the MS series by 1 visit, and panel C shows a series of MS data that precedes the RA series by 1 visit.

Cross-correlation function (CCF) is used to identify the time lag (visit lag in our analysis) of one variable that may be a useful predictor of another variable. The cross-correlation of a pair of random process, also considered as time-dependent Pearson correlation coefficient, is the correlation between values of the processes at different times, as a function of the two times. The background and mathematical form of CCF may be found in most statistical textbooks of time series analysis [22–24]. CCF is a standard statistical tool in time series analysis to estimate the degree to which two series are correlated. Fig 1 provides a graphical illustration of the three possible visit lag scenarios in our study, with visit lags of 0 (panel A), −1 (panel B), and +1 (panel C). CCF assesses the correlation between RA and MS series at all visit lags, which enables us to characterize their longitudinal relationship and to identify the lag at which the RA and MS series have the strongest correlation. When the strongest correlation was identified at visit lag 0, this indicates that the RA and MS series evolved simultaneously over time. When the strongest correlation was identified at visit lag −1, this indicates that the relationship between the two series was strongest (i.e. the two series are most similar in their trend of series) when RA preceded MS by 1 visit. Similarly, when the strongest correlation was identified at visit lag +1, this indicates that the relation of two series was strongest when MS preceded RA by 1 visit.

**Fig 2:**
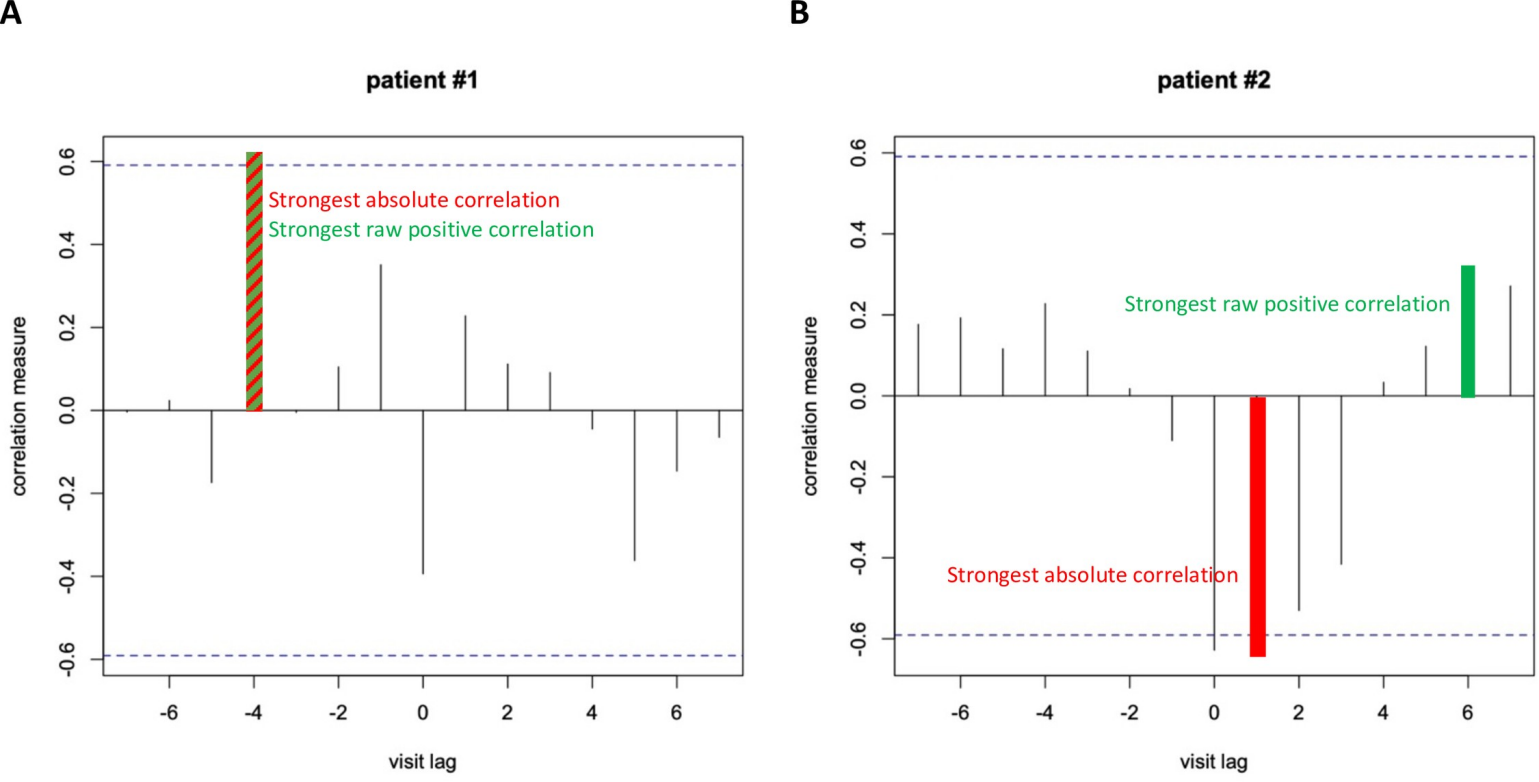
Examples of CCF plots of RA on MS for two patients included in this study. The conventional CCF identified the strongest absolute correlation (tallest overall spike illustrated in red and green stripes the same as the strongest positive correlation) at lag = −4 (panel A) and +1 (panel B). The modified CCF identified the strongest positive correlation (tallest spike above the zero line) at lag = −4 (panel A, in red and green stripes because the same as the strongest absolute correlation) and +6 (panel B, illustrated in green). The dashed blue horizontal lines indicate statistical significance at the p = 0.05 level.

The conventional CCF identifies the strongest absolute correlation and thus allows the selection of visit lags with negative correlations (e.g. worsening structural parameter and improving functional parameter, or vice versa). For glaucoma, negative correlations are not expected across the full spectrum of the disease, because worsening on both structure and function should eventually occur. We therefore modified the conventional CCF and forced it to only identify visit lags with positive correlations. Of note, a preliminary analysis of the longitudinal trend for structural and functional series showed that 9 patients had a tendency towards improvement on both structure and function, but this tendency was statistically significant on only either the structural or functional parameter. While a mild learning effect may account for improvement in the six patients that improved on function, spurious variability may partly explain the structural improvement observed in three patients as surgical procedures to lower intra-ocular pressure was ruled out. The difference between the conventional and modified CCF is illustrated in Fig 2, which shows CCF graphs for two patients enrolled in the study as examples. The vertical lines (spikes) indicate the strength of the correlation measured at each visit lag. The dashed blue horizontal lines indicate statistical significance at the p = 0.05 level. For the patient shown in panel A of Fig 2, both the conventional CCF and the modified CCF identified visit lag −4 as having the strongest correlation (the green and red spikes overlap). For the patient shown in panel B of Fig 2, however, there is a discrepancy between the results of the conventional and modified CCFs. The conventional CCF identified the strongest absolute correlation (shown in red) at visit lag +1, while the modified CCF identified the strongest positive correlation (shown in green) at visit lag +6. To ensure that the visit lags were not due to randomness or to measurement error, we calculated the percentage of patients whose visit lag at the strongest and the 2^nd^ strongest absolute or positive raw correlation had the same sign.

For both CCFs, the sign of the visit lag with the strongest correlation was used to categorize patients into three groups: a) RA and MS series evolved simultaneously (lag = 0), b) RA preceded MS series (lag<0), and c) MS preceded RA series (lag>0). The mean visit lag for each group was reported as a descriptive statistic and was rounded down to the nearest whole number to be used in the analysis of longitudinal lagging relationship. In order to examine whether significant variations exist in the longitudinal lagging relationship at the subject level, the lagging relationship was assessed within each of the three groups. Treating the RA measure at the mean visit lag as the response variable or predictor, linear model with restricted maximum likelihood approach (REML), and linear mixed effect model with a random effect term to account for within-patient correlation were fitted for each group. Likelihood ratio test (LRT) was applied to examine the difference between the model with and without random effect from subjects in each group. The difference in strongest correlation among lag groups was assessed using the Kruskal-Wallis test as a non-parametric approach. All analyses were carried out in R 3.6.0 [25].

## Results

**Fig 3:**
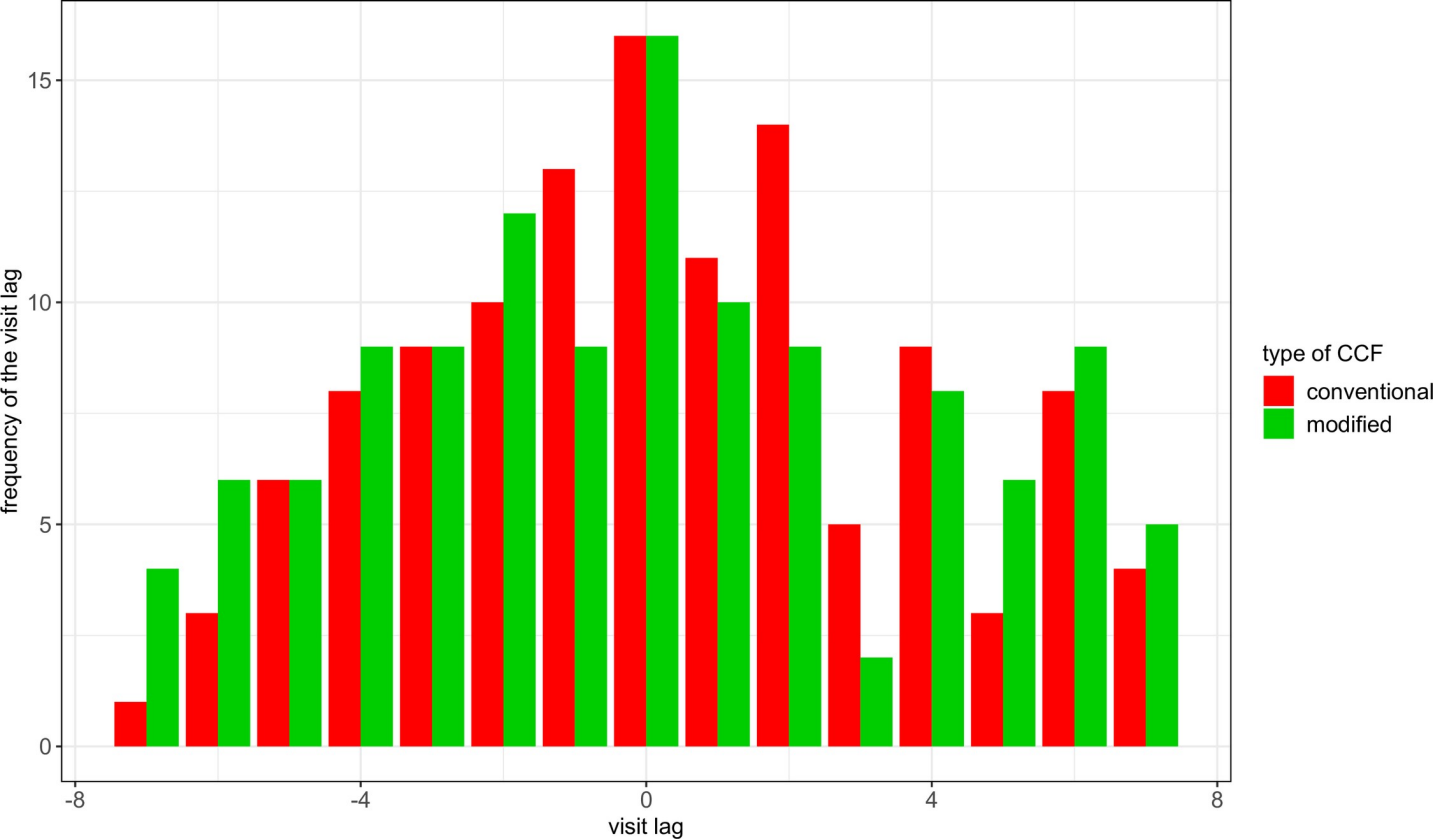
The number of patients with the strongest correlation at each visit lag for the RA on MS relation using the conventional and modified CCFs. Negative lags indicate that RA precedes MS series and positive lags indicate that MS precedes RA series. With both CCFs, the number of visit lags ranges from −7 to 7.

The number of visit lags for all patients with the conventional and modified CCFs are summarized in Fig 3. While for most patients the RA and MS series evolved simultaneously (lag = 0), the number of visit lag ranged from −7 to +7 for both CCFs. With the conventional CCF, of the 50 patients who had their strongest correlation at a negative visit lag, 58% (29 patients) also had their 2^nd^ highest correlation at a negative visit lag (another 4 patients had their 2^nd^ highest correlation at lag = 0). Of the 54 patients who had their strongest correlation at a positive visit lag, 56% (30 patients) had their 2^nd^ highest correlation at a positive visit lag (another 10 patients had their 2^nd^ highest correlation at lag = 0). With the modified CCF, of the 55 patients who had their strongest correlation at a negative visit lag, 45% (25 patients) also had their 2^nd^ highest correlation at a negative visit lag (another 5 patients had their 2^nd^ highest correlation at lag = 0). Of the 49 patients who had their strongest correlation at a positive visit lag, 45% (22 patients) had their 2^nd^ highest correlation at a positive visit lag (another 2 patients had their 2^nd^ highest correlation at lag = 0).

**Fig 4:**
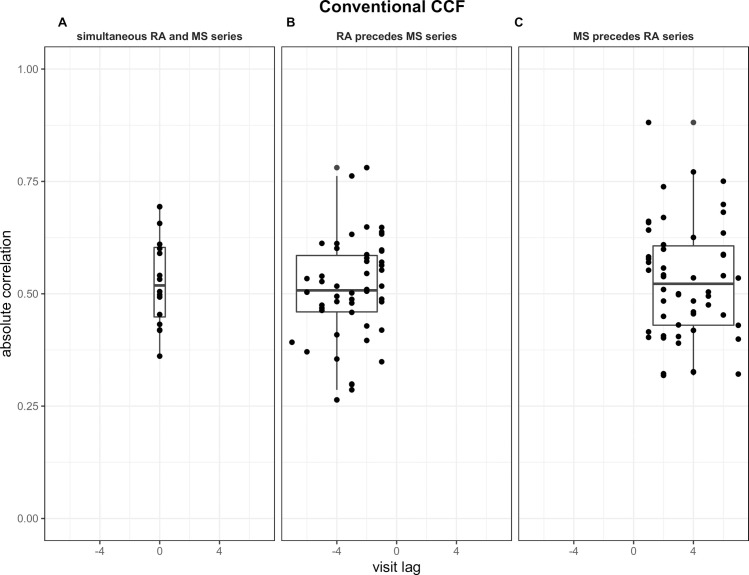
Boxplots of measure of the strongest absolute correlations at different group of visit lags using the conventional CCF. Boxplots are shown for the visit lag = 0 in which the RA and MS series evolve simultaneously (Panel A), visit lag<0 in which change in the RA series precedes change in the MS series (Panel B), and for visit lag>0 in which change in the MS series precedes change in the RA series.

**Fig 5:**
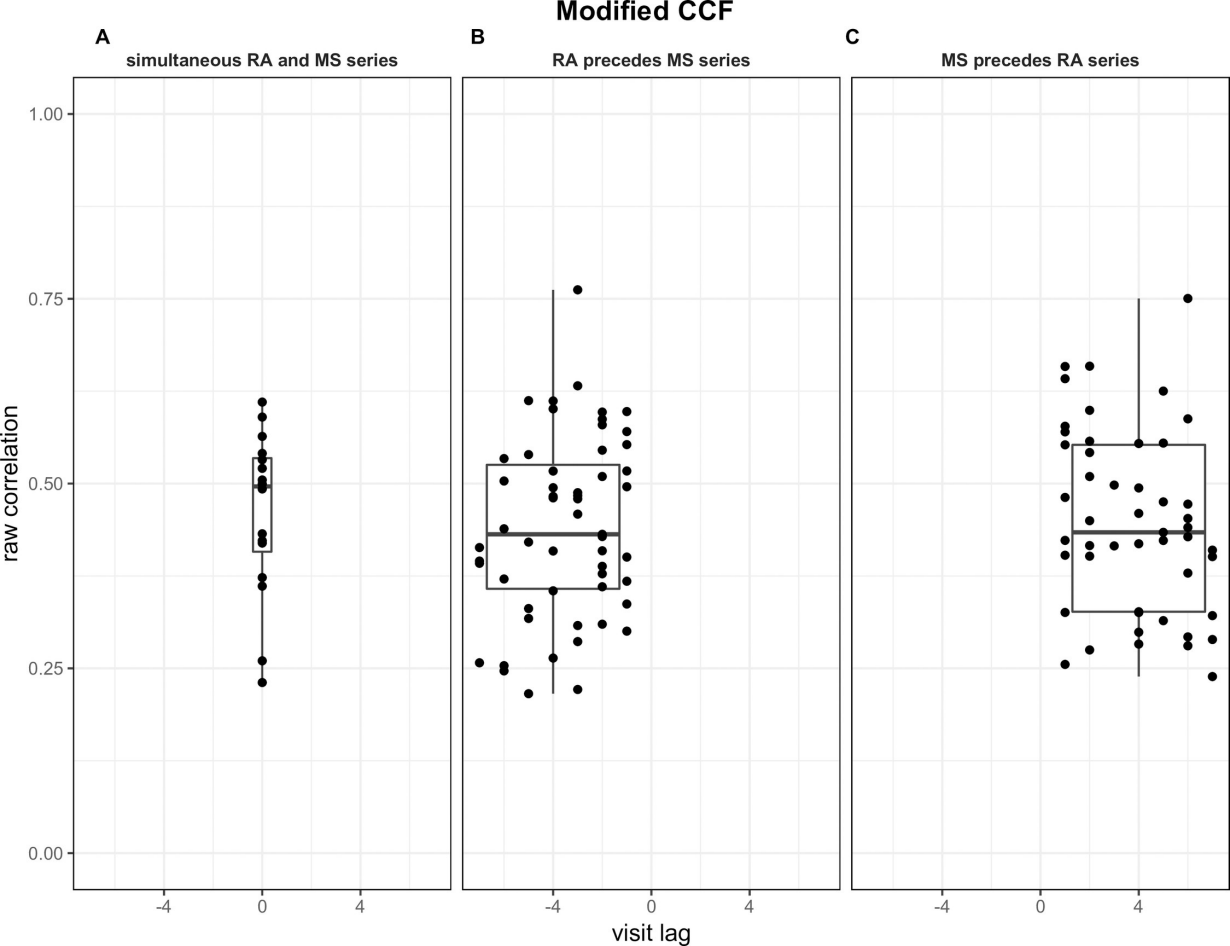
Boxplots of measure of the strongest positive correlations at different group of visit lags using the modified CCF. Boxplots are shown for the visit lag = 0 in which the RA and MS series evolve simultaneously (Panel A), visit lag<0 in which change in the RA series precedes change in the MS series (Panel B), and for visit lag>0 in which change in the MS series precedes change in the RA series.

Fig 4 summarizes the strongest absolute correlation obtained against the correspondent visit lag for patients obtained using the conventional CCF. Fig 5 summarizes the strongest positive correlation against the correspondent visit lag for patients obtained with the modified CCF. Panels A, B, and C in both Figs illustrate the results obtained for patients in each of the three visit lag groups. Table 2 summarizes the descriptive statistics for each group with both CCFs. The number of patients and standard deviation of the correlation for each group were relatively close for both CCFs. While the overall mean correlations were stronger for the conventional CCF than for the modified CCF, the range of correlation was similar, spanning from 0.26 to 0.88 and 0.22 to 0.76, respectively. While the correlations had a tighter range in patients with a lag = 0 (panel A, Figs 4 and 5), no systematic pattern between visit lags and the respective correlation measures was observed for patients with lag<0 and lag>0 with both CCFs (panels B and C, Figs 4 and 5). For the conventional CCF, the median strongest absolute correlations are very close for all three groups (*p* = 0.78). For the modified CCF, the median strongest positive correlations are also very close for all three groups (*p* = 0.75).

**Table 2:** Summary of descriptive statistics for each visit lag group with the use of conventional and modified CCFs.

	Conventional CCF				Modified CCF			
	N (eyes)	Mean lag	Mean Cor (SD)	Cor range	N (eyes)	Mean lag	Mean cor (SD)	Cor range
Lag = 0	16	0	0.53 (0.10)	(0.36, 0.69)	16	0	0.46 (0.11)	(0.23, 0.61)
Lag<0	50	−2.94	0.51 (0.11)	(0.26, 0.78)	55	−3.45	0.44 (0.12)	(0.22, 0.76)
Lag>0	54	3.35	0.53 (0.13)	(0.32, 0.88)	49	3.78	0.45 (0.12)	(0.24, 0.75)

CCF: Cross-correlation function; N: Number of eyes; Cor: Correlation.

Table 3 summarizes the results obtained with LRT to determine whether the lagging relationship between the structural and functional series varied within each lag group. The variation of the lagging longitudinal relation within each group was found to be significant (all p-values <0.0001). This was observed for both the conventional and modified CCFs.

**Table 3:** Result of LRT to compare the models with and without random effect for within three visit groups.

		Conventional CCF			Modified CCF		
	Model	RA as predictor					
	random effect	Log likelihood	LR	p-value	Log likelihood	LR	p-value
Lag = 0	With	−783.32			−755.15		
	Without	−873.24	179.85	<0.0001	−862.56	214.82	<0.0001
Lag<0	With	-1997.35			-1933.10		
	Without	-2231.70	468.71	<0.0001	-2210.24	554.29	<0.0001
Lag>0	With	-1916.74			-1778.13		
	Without	-2163.43	493.39	<0.0001	-1954.98	353.71	<0.0001
		**RA as response variable**					
Lag = 0	With	−606.73			−590.95		
	Without	−784.12	354.78	<0.0001	−788.86	395.80	<0.0001
Lag<0	With	-1545.64			-1532.20		
	Without	-2072.74	1054.19	<0.0001	-2068.40	1072.41	<0.0001
Lag>0	With	-1466.36			-1279.66		
	Without	-2018.94	1105.17	<0.0001	-1795.21	1031.09	<0.0001

CCF: Cross-correlation function; RA: Rim area; LR: Likelihood ratio.

## Discussion

The relationship between structure and function is complex in glaucoma, with imperfect associations reported even in histologic data from human eyes [26–28]. It is therefore unsurprising that relatively low correlations have been reported between structural and functional measures in clinical studies. Nassiri et al. [5] suggested that a possible time lag between structural and functional change may be in part responsible for the weak cross-sectionals associations between structural and functional measurements. In the present study, we systematically characterized and quantified the relationship between longitudinal series of structural and functional data across patients. Our results also suggest that the temporal relationship between detectable structural and functional change may follow different patterns in different glaucoma patients. Differences were observed in three aspects: the number of visit lags that maximizes the structure-function relationship, the strength of the correlation, and the variation of the lagging relation within each visit lag group.

The temporal relationship between structural and functional changes can be effectively assessed with CCF in the context of time series analysis. In the present study, 13% (n = 16) of the patients had their strongest correlation when no time lag was introduced, suggesting that structural and functional change was detected simultaneously. This percentage is consistent with data reported in the OHTS [11] and EMGT [29] studies, in which a little over 10% of eyes reached the structural and functional endpoints simultaneously, and is in general agreement with the findings of cohort studies in eyes with ocular hypertension [9, 10] and POAG [6–8]. For the remaining 87% (n = 104) of the patients, the strongest correlation between the structural and functional series was observed at either positive or negative lags. This finding is at odds with the common belief that imaging techniques are more sensitive to structural loss in early disease compared to visual field tests are to functional loss, resulting in earlier structural changes. Data from large randomized clinical trials as well as observational cohort studies have shown, however, that this is not the case [6–12, 29]. Consistent with these studies, our results show that for 54 (45%) (conventional CCF) and 49 (41%) (modified CCF) patients, the strongest structure-function correlation was obtained when the functional series preceded the structural series (positive lag). This suggests that in these patients, the detection of functional change may have preceded the detection of structural change. While this may be counter-intuitive, there is mounting evidence suggesting that structurally intact retinal ganglion cells may be dysfunctional [30]. Research geared towards identifying predictive factors will allow for early intervention in patients with structurally intact but dysfunctional retinal ganglion cells.

**Fig 6:**
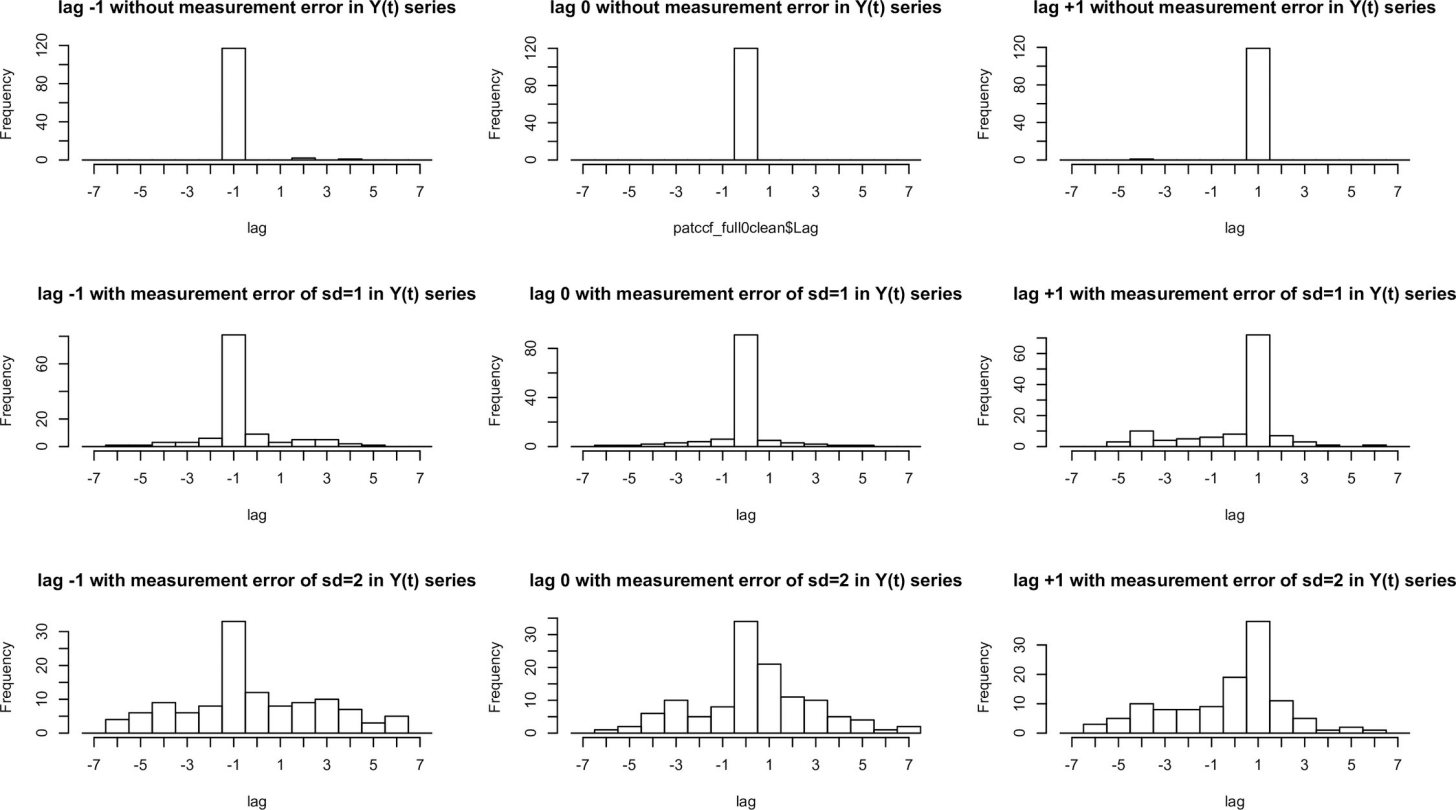
Histograms of lags for 120 pairs of 11 series in 9 simulated datasets with different parameter settings. An autoregressive model (AR = 1) with decreasing trend (0.5) and random error with Gaussian process of mean 0 and SD 1 was used to simulate the first series X(t); the second series, Y(t), was simulated by scaling (0.8) on X(t) series with lag -1,0 and 1 for panels in column 1, 2 and 3, respectively. While the panels in row 1 indicate the result of no added random error to Y(t) series, random error with Gaussian process of mean 0 and SD 1, mean 0 and SD 2 was further added in to the Y(t) series for the panels in row 2 and 3.

Similar to the Pearson correlation coefficient, the CCF is limited in that it does not filter out the measurement errors. When the data is free of measurement errors, the CCF identifies the signal between two series precisely. However, with the presence of measurement errors, the actual trend of signal obtained using CCF will be affected by the trend of measurement errors. The weaker the signal relative to the measurement error, the more variation will be introduced to the observed visit lags and correlation. To understand how the presence of measurement errors may influence the identified visit lags by CCF, 9 scenarios were simulated for 120 pairs of 11 series to provide an overview of the potential impact of measurement errors in this study. An autoregressive model (AR(1)) with decreasing trend (0.5) and random error with Gaussian process of mean 0 and standard deviation (SD) 1 was used to simulate the first series X(t); the second series, Y(t), was simulated by scaling (0.8) on X(t) series with lag -1,0 and 1 for panels in column 1, 2 and 3 of Fig 6, respectively. While the panels in row 1 indicate the result of no added random error to Y(t) series, random error with Gaussian process of mean 0 and SD 1, mean 0 and SD 2 was further added in to the Y(t) series for the panels in row 2 and 3. With the presence of random errors of SD 1 in both series, CCF accurately identified 68%, 76%, 60% of lag of -1, 0 and +1 between two series. Even with the presence of random errors of SD 1 in X(t) series and SD 2 in Y(t) series, CCF still identified around half of them within 1 lag from the true lag (43%, 53% and 57% for lag of -1, 0, and +1). Though the structure and function series in glaucoma data may contain some measurement error, we consider that the variation of the identified visit lags of our study (Fig 3) is large and likely attributed to the variation of the true lags between series, and not simply the result of measurement errors.

Overall, we obtained similar results with the conventional and modified CCFs. While the range of mean and standard deviation of the strongest absolute correlation were similar across three groups, the use of the modified CCF lowered the mean correlation compared to the conventional CCF. The mean correlation of the conventional CCF may have been inflated because it accounts for either the most positive or negative correlation. Using the modified CCF, when the visit lag with the strongest correlation was negative, it was replaced by highest positive correlation, which by design had a weaker absolute correlation measure. Customizing the conventional CCF was important because the latter provides a more appropriate and informative quantitative profile for our study.

The quantitative profile of the temporal relationship between structural and functional measurements has been extensively discussed [31, 32] and has implications for models developed to identify glaucoma progression. Our finding supports the previously reported existence of individual differences in the temporal relationship between structural and functional change [5, 6, 33, 34]. Approaches that seek to identify progression using only structure or function are therefore likely to miss early changes is some patients. Similarly, models that use a Bayesian approach to predict function based on a structural prior [35, 36] would be expected to perform well for patients in which structural loss detected prior to functional loss. But their performance would be hampered for patients in which functional change can be detected first. In contrast, progression models based on both structural and functional parameters may identify progression in patients with various temporal patterns of progression [37, 38].

Significant efforts have been devoted to improving the strength of the structure-function relationship, including expressing structural and functional data in similar units [1], using potentially more sensitive structural [2, 3] and functional [39] tests, and focusing on specific sectors [2] or on the macular region [40]. While these efforts have resulted in modest improvements, the associations between structure and function remain weak. Hood et al. [41] showed that when the displacement of the retinal ganglion cells [42] is taken into account, retinal nerve fiber layer and visual field defects within the central ten degrees can be compared directly. Other researchers have sought to improve the structure-function relationship by improving the mapping of the optic nerve head to the visual field locations [43]. This customized approach based on anatomical features such as the position of the optic nerve head and raphe and the axial length, led to important shifts in approximately 12% of the general population. Such individualized methods may ultimately be necessary in order to optimize the assessment of change in glaucoma patients.

Several limitations need to be understood when interpreting the results of this study. First, our report of CCF aims to serve as descriptive statistics in providing an overview of the relationship between structure and function series. Therefore, the visit lag with the strongest correlation was not required to reach statistical significance. In some patients, the strongest correlation could therefore have been relatively weak and relatively similar to the correlations at other visit lags. Overall, the sign of the 2^nd^ strongest correlation was similar to that of the strongest correlation. Second, a limitation inherent to the CCF is that the correlations at larger visit lags were computed using fewer data points from the longitudinal series, compared to the correlations at smaller visit lag. As the series are increasingly shifted, a smaller number of visits overlap. The correlations at different visit lags were therefore derived using unequal amounts of data, which creates a different variation base in the estimation of the correlation. In this study, we minimized the impact of this limitation, by including relatively long follow-up series. Third, the changes in both the RA and MS series could be due to glaucoma progression, aging effect, or a combination of both. As a result, the lag in the longitudinal relationship between the two series reflects both effects. Ideally, we would use age-corrected data to tease out the impact of age on the data, but age correction was not available for the RA data. Fourth, the potential difference in interindividual variability between structure and function measures and the floor effect of structure measures were not addressed in this study. As the primary interest of this study was to characterize the relation between these two series, CCF serves as a simple descriptive statistical measure to summarize data structure. More advanced statistical tools (e.g. linear mixed effects model) are anticipated to use in order to accommodate these issues in our future research. Fifth, because visual field testing is more susceptible to cataracts than imaging, it is possible that lens opacification in part explain the detection of functional change prior to structural change. Sixth, the use of visit as a time unit could result in a biased correlation measure. While the interval between each visit was not identical for all patients and all visits, testing was done at fairly regular intervals in the DIGS and ADAGES studies. The mean interval length between visits for all patients included in this study was 9.5 months (range of mean interval 7.3–11.9) and the standard deviation of the mean visit interval for each patient was 1 month. The use of visit as a measurement unit therefore had a negligible impact on our results because of the limited variability among visit intervals. Lastly, the structural measures used in this study are limited by the utilization of the HRT II which while being a robust optic disc imaging device remains a two-dimensional imaging device that lacks the possibilities of modern high-resolution OCT systems.

In summary, the temporal relationship between longitudinal and functional data in glaucoma was characterized and quantified using cross-correlation functions. We found that in different patients, the strongest correlation can occur either when there is no lag, or when a positive or negative lag is introduced. This suggests that while structural and functional defects develop simultaneously in some patients, in others, either structural or functional defects can be detected first. The results of our study have implications for the clinical management of glaucoma patients as they indicate that both structure and function should be monitored closely for change. Our findings also support the inclusion of both structural and functional parameters in progression models. Our results suggest that progression should be considered at subject level because the detection of change is not similar for all patients. An individualized dynamic model utilizing both structure and function may provide clinicians more information about progression.

## Supporting information

S1 File Dataset for 120 eyes containing the baseline data. (CSV)

S2 File Dataset for 120 eyes containing the longitudinal structural and functional data. (CSV)
